# Biosynthetic Pathways and Functions of Indole-3-Acetic Acid in Microorganisms

**DOI:** 10.3390/microorganisms11082077

**Published:** 2023-08-12

**Authors:** Jintian Tang, Yukang Li, Leilei Zhang, Jintao Mu, Yangyang Jiang, Huilan Fu, Yafen Zhang, Haifeng Cui, Xiaoping Yu, Zihong Ye

**Affiliations:** Zhejiang Provincial Key Laboratory of Biometrology and Inspection & Quarantine, College of Life Sciences, China Jiliang University, Hangzhou 310018, China; jintiantang@cjlu.edu.cn (J.T.); zll18815563299@163.com (L.Z.);

**Keywords:** indole-3-acetic acid, biosynthetic pathways, microorganisms, auxin

## Abstract

Indole-3-acetic acid (IAA) belongs to the family of auxin indole derivatives. IAA regulates almost all aspects of plant growth and development, and is one of the most important plant hormones. In microorganisms too, IAA plays an important role in growth, development, and even plant interaction. Therefore, mechanism studies on the biosynthesis and functions of IAA in microorganisms can promote the production and utilization of IAA in agriculture. This mini-review mainly summarizes the biosynthesis pathways that have been reported in microorganisms, including the indole-3-acetamide pathway, indole-3-pyruvate pathway, tryptamine pathway, indole-3-acetonitrile pathway, tryptophan side chain oxidase pathway, and non-tryptophan dependent pathway. Some pathways interact with each other through common key genes to constitute a network of IAA biosynthesis. In addition, functional studies of IAA in microorganisms, divided into three categories, have also been summarized: the effects on microorganisms, the virulence on plants, and the beneficial impacts on plants.

## 1. Introduction

IAA is an important plant hormone belonging to the auxin family of indole derivatives. It is the most abundant and fundamental naturally occurring plant hormone that controls almost every aspect of plant growth and development, such as cell division, elongation, fruit development, and senescence [1,2]. It can also increase plant protection against external stress [3]. IAA can be synthesized not only in plants but also in many microorganisms that interact with plants, including bacteria and fungi [4,5].

Microbial biosynthesis of IAA can be classified into tryptophan-dependent and tryptophan-independent pathways based on whether tryptophan (Trp) is used as a precursor. These pathways produce IAA with some similarities to that of plants [6,7,8]. In the tryptophan-dependent pathways of microorganisms, there are different intermediate metabolites, and current research has roughly divided them into five pathways: the indole-3-acetamide pathway (IAM), the indole-3-pyruvic acid pathway (IPA/IPyA), the indole-3-acetonitrile pathway (IAN), the tryptamine pathway (TAM), and the tryptophan side-chain oxidase pathway (TSO). L-tryptophan, the key precursor for IAA biosynthesis in the tryptophan-dependent pathway, is a relatively rare amino acid that is the most energy-dense of all amino acids. The synthesis cost for microbial cells is high, so the endogenous tryptophan concentration in microorganisms is often low. High concentrations of IAA are only produced when an excess of exogenous tryptophan is supplied [9]. The IAM and IPA pathways are the two most common biosynthetic pathways for IAA in microorganisms. In addition, it has been found in very few microorganisms that IAA is synthesized through tryptophan-independent pathways, which mainly use indole-3-glycerol phosphate or indole as the main precursors. However, this pathway has not been studied in-depth, and the key enzymes and genes involved have not been confirmed [6].

In addition to regulating their physiological functions and adapting to external stress and microbial–microbial communication, IAA produced by microorganisms can often participate as a signaling molecule in the interaction between microorganisms and plants, regulating plant growth and development, and causing physiological and pathological changes in plants [8,10,11,12,13,14]. Therefore, it is of great significance to clarify the IAA biosynthesis pathways in microorganisms for studying the synthesis regulation of microbial IAA and the functions of IAA.

## 2. Biosynthetic Pathways of IAA in Microorganisms

### 2.1. The IAM Pathway 

**Figure 1:**
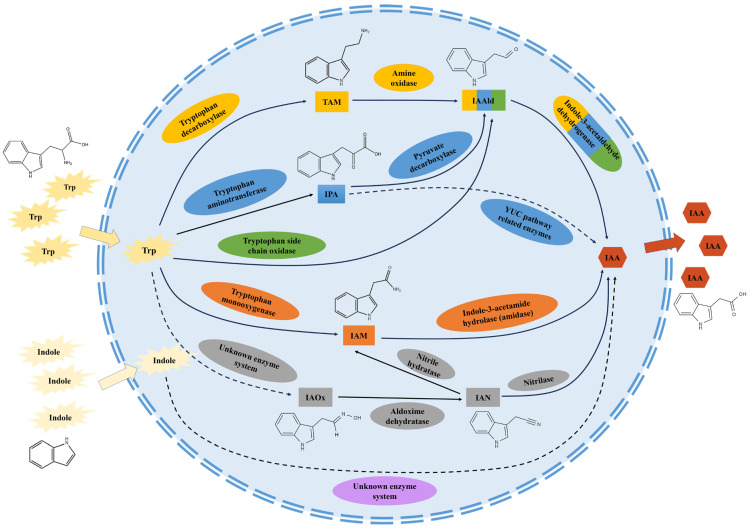
The tryptophan and non-tryptophan dependent auxin biosynthesis pathways of microorganisms found in current research. Blue dashed circle: microbial cells; black solid pointed tip: known IAA microbial synthesis pathway; black dashed tip: unknown IAA microbial synthesis pathway; explosive: IAA synthetic precursor; square: IAA synthetic intermediate; hexagonal: IAA; oval shape: IAA synthesis-related enzymes; yellow: TAM pathway; blue: IPA pathway; green: TSO pathway; orange: IAM pathway; grey: IAN pathway; and purple: non-tryptophan-dependent pathway.

The IAA biosynthesis pathway through IAM has been extensively studied. In this pathway, tryptophan is first converted to IAM by tryptophan monooxygenase, followed by hydrolysis of IAM to IAA and ammonia by indole-3-acetamide hydrolase (Figure 1). In microorganisms, two key genes, *IaaM* and *IaaH*, encoding tryptophan monooxygenase and indole-3-acetamide hydrolase, respectively, have been first identified in *Pseudomonas savastanoi* [15]. Subsequently, homologous genes of *IaaM* and *IaaH* have been identified on the T-DNA of *Agrobacterium tumefaciens*, which can co-regulate IAA biosynthesis from tryptophan. The co-transcription of *IaaM* and *IaaH* on the T-DNA of *A. tumefaciens* can lead to the overproduction of IAA in host plants during infection, ultimately resulting in the formation of plant tumors [8,16,17,18]. In addition, the IAM pathway has also been identified as the major pathway for IAA biosynthesis in the anthracnose fungi, *Colletotrichum gloeosporioides*, *C. acutatum*, and *C. fructicola* [19,20,21]. *C. fructicola* has been found as an endophytic fungus in *Coffea arabica*. The detected activity of tryptophan 2-monooxygenase shows the existence of the IAM pathway in *C. fructicola*. And the extracted crude IAA of *C. fructicola* can stimulate the coleoptile elongation of maize, rye, and rice [19]. For actinomycetes, endophytic *Streptomyces* sp. shows evident growth-promoting effect on medicinal plants species *Taxus chinensis* and *Artemisia annua*. High performance liquid chromatography (HPLC) and genetic analysis have detected IAM and screened homologous gene of *IaaM*, indicating the existence of the IAM pathway in *Streptomyces* sp. [22]. Current studies have showed that the IAM pathway exists in most species of bacteria and some species of other microorganisms including fungi, actinomycetes, and cyanobacteria [20]. This pathway is one of the most well-studied tryptophan-dependent pathways.

### 2.2. The IPA Pathway

The IPA pathway was first discovered in plants and is also one of the major pathways for microbial IAA biosynthesis. In this pathway, tryptophan is first converted to IPA by aminotransferases and then to indole-3-acetaldehyde (IAAld/IAD) via the action of the pyruvate decarboxylase (IPDC). Finally, IAAld is converted to IAA via the action of aldehyde dehydrogenases. In the IPA pathway, the *TAM* gene encodes the aminotransferases, the *IPDC* gene encodes the decarboxylase, and the *IAD* gene encodes the aldehyde dehydrogenase [8] (Figure 1).

Numerous studies have showed that the IPA pathway is a major pathway for IAA biosynthesis that is widely present in bacteria [23,24,25,26]. In *Azospirillum brasilense*, disruption of key enzymes in the IPA pathway leads to a significant reduction in IAA production, which suggests that the IPA pathway is a major IAA biosynthesis pathway [27]. The *IPDC* homologous gene has been identified in *Bacillus thuringiensis*, and the *IPDC* deletion mutant showed a significant reduction in IAA synthesis in the presence of tryptophan. Although there is no significant difference in growth between *IPDC* deletion mutants and wild type, mutants lost their ability to colonize maize roots and promote plant growth [28]. The *IPDC* homologous genes have also been identified in a range of strains of *Enterobacter* sp. via genome analysis and RT-qPCR, indicating that the IPA pathway is involved in IAA biosynthesis in these bacterial strains [29].

For fungi, the IPA pathway is also considered to be the major pathway for IAA biosynthesis [30,31]. A series of enzymes involved in the IPA pathway have been identified in the corn smut fungus *Ustilago maydis*, including tryptophan aminotransferases (TAMs) Tam1 and Tam2, which convert tryptophan to IPA [8], and indole-3-aldehyde dehydrogenases (IADs), Iad1 and Iad2, which convert IAAld to IAA [30,32]. The transaminase Tam1/Aro8 homologous gene *SsAro8* of the IPA pathway has been identified in the sugarcane smut fungus *Sporisorium scitamineum*. The *SsAro8* deletion mutant is defective in IAA biosynthesis, oxidative stress tolerance, binuclear hyphae formation, biofilm formation, and pathogenicity [33,34,35]. *Magnaporthe oryzae* has been found to generate IAA in its hyphae and conidia. Genetic analysis of *M. oryzae* shows a complete IPA pathway, including tryptophan aminotransferase (MoTam1) and indole-3-pyruvic acid decarboxylase (MoIpd1). *MoTam1* or *MoIpd1* gene deletion mutants shows varying degrees of defects in IAA biosynthesis, hyphal growth, conidiation, and pathogenicity of *M. oryzae*. The targeted metabolomic analysis further reveals the existence of an IPA pathway catalyzed by MoIpd1, which contributes to IAA production in *M. oryzae* [36]. In the mushroom *Lentinula edodes*, the expression of an aldehyde dehydrogenase gene *ald1* has been detected that is highly induced by IAAld in roots, which suggests the existence of the IPA pathway [31]. In addition, some species of ectomycorrhizal fungi (ECM) have been found to produce IAA in liquid medium with L-tryptophan. The tryptophan aminotransferase activity and HPLC detection indicate that these ECMs synthesize IAA through the indole-3-pyruvic acid pathway [37,38]. In summary, the IPA pathway is a major pathway for IAA biosynthesis and is widely present in bacteria and fungi.

### 2.3. The TAM Pathway

The TAM pathway is one of the four tryptophan-dependent pathways of IAA biosynthesis in plants and is also reported in microorganisms. The process of the TAM pathway is similar in both plants and microorganisms. Tryptophan is first converted to tryptamine by tryptophan decarboxylase, and then tryptamine is further converted to indole-3-acetaldehyde by amine oxidase. Finally, IAAld is converted to IAA by aldehyde dehydrogenase (Figure 1).

In microorganisms, the tryptamine decarboxylase was first identified in *B. cereus*, with the production of tryptamine when *B. cereus* was treated with tryptophan [39]. In the basidiomycete fungi *Rhodosporidiobolus fluvialis*, an intermediate metabolite (tryptamine) of the TAM pathway was detected using LC-MS. Further studies showed the activity of tryptophan decarboxylase, which revealed the presence of the TAM pathway in *R. fluvialis* [40]. *Metarhizium robertsii* identified a tryptophan decarboxylase (MrTDC) homolog of *Catharanthus roseus*. The lack of MrTDC was resulted in the defective conversion of tryptophan to tryptamine and affected the production of IAA in *M. robertsii* [41].

Compared with the IAM and IPA pathways, the TAM pathway is still unclear in microorganisms since the key amine oxidase in the conversion of tryptamine to IAAld is rarely reported. Thus, it has been inferred that the TAM pathway generally coexists with other IAA biosynthesis pathways to produce IAA in microorganisms.

### 2.4. The IAN Pathway

Research on the IAN pathway is mostly focused on plants. Tryptophan is first converted by cytochrome P450 enzymes into indole-3-acetaldoxime (IAOx). IAOx is then directly converted into IAN, or converted into indole-3-acetyl glucosinolate and then into IAN. Finally, IAN is converted into IAA by nitrilase, which is a key enzyme in this pathway [42,43,44] (Figure 1).

Studies on the IAN pathway in microorganisms are still limited. Although the key enzyme for the conversion of tryptophan to IAOx has not been identified in microorganisms, the presence of several aldoxime dehydratases (Oxds) that convert aldoximes to nitriles have been confirmed in *Bacillus* sp. The activity of indole-3-acetaldehyde oxime dehydratase has been detected in *Sclerotinia sclerotiorum* and *Bradyrhizobium* sp. Studies show that Oxds genes are always coexisting with genes encoding nitrile hydrolases or nitrile hydratases [45,46,47,48]. Therefore, it indicates the existence of the IAN (IAOx-IAN) pathway in microorganisms [27]. In *Variovorax boronicumulans*, a bacterium with a growth-promoting effect on plants, tryptophan cannot be used as a starting material in the synthesis of IAA, whereas IAN can be used as a precursor to synthesize IAA. Genome analysis shows the presence of *NitA* and *IamA* related to the IAN pathway in *V. boronicumulans*. Overexpression of the *NitA* and *IamA* genes shows that NitA has nitrilase activity, while IamA has amidase activity. Therefore, it is speculated that *V. boronicumulans* has two enzyme systems for the IAN pathway with different regulatory mechanisms: a nitrilase system and a nitrile hydratase/amidase system. In the nitrilase system, IAN is quickly converted to IAA for cell growth through nitrilase, while in the nitrile hydratase (NHase)/amidase system, IAN is first converted to IAM and then to IAA slowly and continuously [49]. In *Bacillus amyloliquefaciens*, the loss of the nitrilase gene *yhcX* in the IAN pathway leads to a 50% decrease in IAA production [50]. The existence of the IAN pathway is also reported in *Xylaria* sp., *Leptosphaeria maculans* and *Arthrobacter pascens* by the identification of nitrile hydratase and nitrilase [51,52].

Currently, in microorganisms only some key enzyme genes have been identified in the process from IAN to IAA, while the key enzyme gene that initially converts tryptophan to IAOxis still absent. Thus, a complete construction of the IAN pathway in microorganisms is needed to reveal the process from tryptophan to IAOx [52].

### 2.5. The TSO Pathway

Compared with other tryptophan-dependent pathways, research on the TSO pathway is limited. In this pathway, tryptophan is directly converted into IAAld by side-chain oxidase, and then IAAld is converted into IAA by indole-3-acetaldehyde dehydrogenase. Currently, this pathway has only been reported in *Pseudomonas fluorescens* among microorganisms. The IAA biosynthesis pathway of *P. fluorescens* involves two key enzymes, TSO and tryptophan transaminase. In addition, the ability of IAA synthesis in the TSO pathway is also weaker compared to other IAA biosynthesis pathways including the IAM and IPA pathways [53] (Figure 1). This suggests that the TSO pathway plays a supplementary or regulatory role in some organisms of IAA synthesis.

### 2.6. Non-Tryptophan-Dependent Pathway

Compared with the tryptophan-dependent pathway, the precursor of IAA synthesis in the non-tryptophan dependent pathway is not tryptophan. IAA synthesis in non-tryptophan-dependent pathways is relatively common in plants, but it has been identified in a few species of microorganisms [20]. In the nitrogen-fixing bacterium *Azospirillum brasilense*, it was found that a significant amount of low-radioactivity IAA was still synthesized when treated with isotopically labeled tryptophan, suggesting the existence of a non-tryptophan-dependent pathway [27]. A similar result of isotopically labeled tryptophan treatment in *A. brasilense* has been also observed in the filamentous fungi *Aspergillus flavus* [41]. In *Saccharomyces cerevisiae*, when treated with isotopically labeled tryptophan, it has been found that the aldehyde dehydrogenase gene (*ALD*) deletion mutant loses the ability of tryptophan metabolism, but it can still produce nonradioactive IAA. Thus, the results suggest that a non-tryptophan-dependent pathway may exist in some fungi. However, the mechanism of the non-tryptophan-dependent pathway has not been revealed to date [54]. A recent study of the endophytic fungus *Cyanodermella asteris* has showed that it uses indole as the precursor to start IAA synthesis to bypass tryptophan, which is similar to the non-tryptophan-dependent pathway in plants [8]. But the enzymes related to this pathway are still unknown in microorganisms [55]. Above all, the current study findings suggest that the non-tryptophan-dependent pathway is not the main pathway, but a replenishment pathway for IAA biosynthesis.

## 3. Interactive Effect of Multiple IAA Biosynthetic Pathways in a Microorganism

IAA biosynthesis pathways are classified according to the precursor, intermediate, and key enzymes. These pathways have also been found to form a closely linked redundant IAA biosynthesis network through common precursors, intermediates, and enzymes in the previous studies, which suggest that they do not always exist separately in a microorganism [41] (Table 1).

In *Arthrobacter pascens*, genome analysis showed the existence of aldehyde dehydrogenase genes (*prr* and *aldH*) and acylamidase genes (*aam* and *gatA*). And HPLC-MS also detected intermediates of IAA biosynthesis, including IAM, IPyA, indole-3-lactic acid (ILA), and the enzymatic degradation product of indole-3-ethanol (TOL). This result indicates that the IAM and IPA pathways are involved in IAA biosynthesis in *A. pascens* [52]. In the plant growth-promoting rhizobacterium (PGPR), a type of bacteria inhabits the plant rhizosphere, *Pseudomonas* sp. UW4 interactions between IAM and IAN pathways have been revealed by the characterization of *aim* and *nitrilase* genes [56,57]. Additionally, in some strains of PGPR *Lysinibacillus* spp., genome analysis shows the existence of key genes in both IPA (*IPDC* and *aldH* genes) and TAM (aromatic-L-aminoacid decarboxylase gene) pathways [58]. For the yeast fungi *Rhodosporidiobolus fluvialis*, IPA treatment results in an increase in IAA in the culture supernatant. Furthermore, the activities of tryptophan aminotransferase, tryptophan 2-monooxygenase, and tryptophan decarboxylase have also been observed in cell crude extract. Thus, results suggested the existence of IPA, TAM, and IAM pathways in *R. fluvialis*, and the IPA pathway is the main route of IAA biosynthesis [52]. For filamentous fungi, different combinations of multiple IAA biosynthesis pathways in each species have been revealed via key genes characterizing, intermediate products detecting, and pathways blocking. TAM and IAM pathways have been found in *Metarhizium robertsii* [41]. IPA and IAN pathways have been found in *Leptosphaeria maculans* and *B. amyloliquefaciens* [51,59]. Furthermore, there are even examples of these three pathways existing in one species. IPA, IAM, and TAM pathways have been identified in *Fusarium delphinoides* and *Rhizobium tropici* [60]. In *R. tropici*, the IAM pathway acts as a replenishment pathway of IAA biosynthesis which is active and not affected by TAM and IPA pathway mutations [61]. In the endophytic fungi *Cyanodermella asteris*, the TAM pathway has been identified as the main way of IAA biosynthesis; meanwhile, IPA and IAM pathways work as a supplement [55]. The YUC (yucca gene family encoding flavin monooxygenases) pathway is a branched metabolic pathway that transforms IPA to IAA in plants [28]. Interestingly, two yucca genes homologous to the *Arabidopsis thaliana* YUC pathway genes have been identified in *Magnaporthe oryzae*. Treatment with yucca protein inhibitors yucasin or deletion of two yucca genes in *M. oryzae* can result in defects of mycelial growth, conidiation, and pathogenicity, indicating that *M. oryzae* can also synthesize IAA from IPA directly through this YUC pathway, except through intermediate IAAId [36,52] (Figure 1). Overall, these redundant systems may be able to remedy the situation when a primary biosynthetic pathway fails to produce IAA, thereby preventing the death of organisms from IAA deficiency. In addition, the expression level of *piTam1*, a key gene in the IPA pathway of the endophytic bacterium *Piriformospora indica*, is highly induced during the biotrophic phase of infection process. The silencing of *piTam1* leads to a decrease in the production of IAA and ILA in the strain, while the ability to colonize barley roots is also affected. However, the vegetative growth of *P. indica* in the medium is not significantly affected, indicating that the other two replenishment pathways (TAM and IAM pathways) in *P. indica* may help in providing the IAA [62]. The IPA pathway genes of the plant pathogenic bacterium *Erwinia herbicola* are highly expressed during the saprophytic phase on the leaf surface, while the IAM pathway genes are highly expressed after the bacteria penetrate the leaf, suggesting that this redundant system may also be a microbial adaptation to the environment [63,64].

## 4. The Functions of IAA in Microorganisms

Like many plants, the main function of IAA in microorganisms is growth regulation. More concretely, different concentrations of IAA have both promoting and inhibitory effects on microbial growth. For example, high concentrations (5000 μM) of IAA significantly affect the growth of *Saccharomyces cerevisiae*, while *Ustilago escultenta* is not sensitive to the same concentration [65]. In *Fusarium delphinoides*, low concentrations (0.5, 5, and 50 μM) of IAA can promote its growth, while its growth is significantly inhibited by high concentrations (500 and 5000 μM) of IAA [65]. In *Fusarium graminearum*, growth is inhibited by any concentration of exogenous IAA [66]. In addition, IAA may also act as a signaling molecule regulating metabolism, cellular compartment, and pathogenicity. The IAA-overproducing mutant RD64 of *Rhizobium meliloti* can synthesize more alginates, lipopolysaccharides (LPS), extracellular polysaccharides (EPS), and biofilms [67] to evade the plant defenses and increase their survival rate under environmental stress including drought and low temperature [68]. *Candida tropicalis* synthesizes IAA through the IPA pathway to promote the formation of biofilms, thereby further enhancing its pathogenicity [69]. Mutations of indole-3-neneneba pyruvate decarboxylase gene *y4wf* and oxidase/dehydrogenase gene *tidC* in *Rhizobium tropici* also lead to increased extracellular polysaccharide synthesis and enhanced stress resistance [61]. During the infection process, the insect pathogenic fungus *Metarhizium robertsii* can utilize tryptophan in the insect cuticle layer to produce more IAA, indicating that IAA plays an important role in infection growth. The following study showed that exogenous IAA can significantly increase the formation of appressoria in the infection process of *M. robertsii* [41,70]. However, the intermediate products of IAA play the opposite roles against IAA. In *Candida albicans*, treatment with exogenous indole or IAN does not affect the growth, but significantly inhibits the formation of biofilm and the ability of producing virulence-related filamentous bodies [71,72]. Hyphae growth of *F. graminearum* is inhibited by exogenous TAM and IAN. IAN also affects the branching mode of hyphae, spore germination, and the production of mycotoxins [66].

In addition to acting directly on the growth of microorganisms, IAA can also act as a virulence factor or plant growth-promoting factor in the interaction between plants and microorganisms. The IAA synthesized and secreted by pathogenic microorganisms can induce the expression of its virulence genes, and inhibit plant immunity by loosening cell walls, opening stomata, and suppressing host defense [73]. Studies have showed that plants often enhance their immune response by inhibiting their own IAA synthesis or response pathways [74]. In the interaction between plants and microorganisms, both pathogenic and symbiotic bacteria and fungi can weaken the host’s immune response by synthesizing and secreting IAA or by affecting IAA synthesis and transport in the host plant, leading to local tissue accumulation of IAA [74,75,76,77,78]. During the early infection stage, the expression of IAA synthesis genes is highly induced in both *Ustilago maydis* and maize. Therefore, a large amount of IAA is synthesized in the infection site. And the salicylic acid (SA)-mediated defense response of the host is weakened. Hyphae proliferate rapidly and eventually form tumors [79,80]. *Plasmodiophora brassicae* can induce the conversion of indole-3-acetonitrile to IAA in the infected tissue and root swelling of cruciferous plants. However, the *A. thaliana* mutant which is lacking the key gene for polar IAA transport can inhibit the infection of *P. brassicae* and the swollen root formation [75]. Additionally, accumulation of IAA in rice enhances susceptibility to blast disease [81], while blocking IAA synthesis by overexpression of indole-3-acetic acid amido synthetase gene can help to acquire resistance to blast disease [82]. Overexpressing *CsGH3.1* and *CsGH3.1L* in citrus significantly brings down free IAA levels, thereby reducing the susceptibility of *Xanthomonas citri* subsp. *citri* [83]. Conversely, a few studies have showed a different opinion, suggesting that the increase in IAA level in plants does not affect the defense or even induce the resistance to pathogens. Induced IAA level promotes resistance of rice to rice sheath blight that is caused by *Rhizoctonia solani* [84]. For rice dwarf virus (RDV), exogenous auxin application promoted the degradation of OsIAA10 protein to release OsARF12, thereby activating the defense-related gene *OsWRKY13* to participate in the resistance of RDV [85].

However, many plants symbiotic bacteria and fungi also produce IAA to stimulate various physiological processes in plants, including cell division, elongation, polarity, apex dominance, senescence, flow, and stress response [86,87,88]. Studies have showed that 80% of bacteria in the root environment can synthesize IAA [89]. PGPRs, such as *Enterobacter* sp., *Serratia marcescens*, *Brevibacillus laterosporus*, *Burkholderia phytofirmans*, *Pseudomonas aeruginosa*, *Glomus mosseae*, etc. [90,91,92,93], have been reported that can directly or indirectly affect host plant growth and development by IAA [11]. Mechanisms of growth-promoting effect on plants by some PGPRs have been revealed. Mutants of *B. thuringiensis* with a blocked main IAA biosynthesis pathway had significantly reduced the ability to promote maize growth compared to the wild-type strain [28]. Similarly, IAA production has been significantly reduced when the IAA synthesis gene *IPDC* of *A. brasilense* is knocked out, and the ability to promote the growth of sorghum roots is also significantly weakened [94]. In addition to promoting growth directly through the production of IAA, microorganisms can also promote plant growth by improving the plant resistance to abiotic stresses. Some PGPR strains, which are isolated from alkali soil, can produce IAA to enhance the salt tolerant of maize and wheat seeds during germination [95]. Salt stress induces the ability of IAA biosynthesis in *Acinetobacter pittii*. Thus, the inoculated soybeans with *A. pittii* show a significantly improvement in plant growth under salt stress [96]. The salt-tolerant strains isolated from saline-alkali soil that can promote the growth of upland cotton can produce IAA [97,98,99]. Studies have speculated that these microorganisms can increase the induced systemic resistance of plants including antioxidant enzyme activity, inorganic salt solute accumulation, and ACC deaminase activity in response to environmental stress through IAA [100], which can promote the growth of various plants (forage legume, common ice plant, rice, wheat, tomato, etc.) under salt stress [100,101,102,103,104]. A new species of root endophytic fungus within *Sordariomycetidae*, named CJAN1179, can produce IAA to promote lateral root growth of *A. thaliana* and increase the uptake of nutrients and water resulted in a threefold increase in lateral root number, indicating a significant plant growth-promoting effect. Therefore, CJAN1179 has great potential for widespread application in arid areas [105]. Similarly, a PGPR *Cronobacter* sp. has a promoting effect on maize plant growth under drought stress by inhibiting abscisic acid (ABA) signaling and inducing IAA biosynthesis in a tryptophan-dependent manner [106]. Except bacteria, studies have showed that some fungi strains with high IAA production can also have a significant promoting effect on the plant growth, diosgenin content, and nutritional value of fenugreek [107,108,109,110,111,112].

## 5. Summary

As research progresses, many key enzyme genes involved in auxin synthesis in microorganisms have been identified, and the main IAA biosynthesis pathway has been fully characterized. However, the complex IAA biosynthesis network and the role of IAA in microorganisms are still unrevealed, including interactions between various regulatory genes and key enzyme genes in some synthesis pathways. In the aspect of IAA functions in microorganisms, further exploration is required on the mechanism of interaction between microbially synthesized IAA and plants, such as how microorganisms communicate with plants through IAA-mediated signaling pathways, whether IAA is a virulence factor, whether IAA amplifies virulence effects and what are the effects of IAA on plant defense mechanisms. Elucidating the synthesis, metabolism, transport, and signal transduction pathways of IAA in microorganisms is essential for the rational and adequate utilization of microorganisms to promote plant growth and development, increase crop yields, improve soil conditions, and create significant economic, environmental, and social benefits.

## Figures and Tables

**Table 1:** Identified and speculated IAA biosynthetic pathways in some species that have been studied and reported.

Species	Pathway					
	IAM	IPA	TAM	IAN	TSO	Non-Tryptophan Dependent
*Agrobacterium tumefaciens*	✓					
*Arthrobacter pascens*	✓	✓		✓		
*Azospirillum brasilense*		✓				✓
*Bacillus amyloliquefaciens*		✓	✓	✓		
*Bacillus cereus*			✓			
*Bacillus thuringiensis*		✓				
*Erwinia herbicola*	✓	✓				
*Escherichia* sp.		✓				
*Herbaspirillum aquaticum*		✓				
*Lysinibacillus* spp.		✓	✓			
*Pseudomonas fluorescens*		✓			✓	
*Pseudomonas putida*				✓		
*Pseudomonas* sp.	✓			✓		
*Rhizobium tropici*	✓	✓	✓			
*Serratia marcescens*	✓	✓				
*Variovorax boronicumulans*				✓		
*Aspergillus flavus*						✓
*Astraeus odoratus*	✓					
*Bradyrhizobium japonicum*				✓		
*Candida tropicalis*		✓				
*Colletotrichum acutatum*	✓					
*Colletotrichum fructicola*	✓					
*Colletotrichum gloeosporioides*	✓					
*Cyanodermella asteris*	✓	✓	✓			✓
*Fusarium delphinoides*	✓	✓	✓			
*Fusarium proliferum*	✓					
*Gyrodon suthepensis*		✓				
*Laccaria bicolor*		✓				
*Lentinula edodes*		✓				
*Leptosphaeria maculans*		✓		✓		
*Magnaporthe oryzae*		✓				
*Metarhizium robertsii*	✓		✓			
*Neurospora crassa*		✓				
*Phlebopus portentosus*		✓				
*Piriformospora indica*		✓				
*Pisolithus albus*		✓				
*Pisolithus orientalis*		✓				
*Rhodosporidiobolus fluvialis*	✓	✓	✓			
*Saccharomyces cerevisiae*						✓
*Scleroderma suthepense*		✓				
*Sporisorium scitamineum*		✓				
*Tricholoma vaccinum*		✓				
*Ustilago maydis*		✓				
*Xylaria* sp.				✓		

## Data Availability

Data are contained within the article.
